# Effects of Various Doses of Selenite on Stinging Nettle (*Urtica dioica* L.)

**DOI:** 10.3390/ijerph7103804

**Published:** 2010-10-22

**Authors:** Olga Krystofova, Vojtech Adam, Petr Babula, Josef Zehnalek, Miroslava Beklova, Ladislav Havel, Rene Kizek

**Affiliations:** 1 Department of Chemistry and Biochemistry, Mendel University in Brno, Zemedelska 1, CZ-613 00 Brno, Czech Republic; E-Mails: olga.krystofova@seznam.cz (O.K.); vojtech.adam@mendelu.cz (V.A.); zehnalek@mendelu.cz (J.Z.); 2 Department of Natural Drugs, Faculty of Pharmacy, University of Veterinary and Pharmaceutical Sciences, Palackeho 1-3, CZ-612 42 Brno, Czech Republic; E-Mail: babulap@vfu.cz (P.B.); 3 Department of Veterinary Ecology and Environmental Protection, Faculty of Veterinary Hygiene and Ecology, University of Veterinary and Pharmaceutical Sciences, Palackeho 1-3, CZ-612 42 Brno, Czech Republic; E-Mail: beklovam@vfu.cz (M.B.); 4 Department of Plant Biology, Faculty of Agronomy, Mendel University in Brno, Zemedelska 1, CZ-613 00 Brno, Czech Republic; E-Mail: lhavel@mendelu.cz (L.H.)

**Keywords:** graphite furnace atomic absorption spectrometry, high performance liquid chromatography with electrochemical detection, plant tissues, heavy metal, plant, nettle, selenium

## Abstract

The aim of this study was to investigate the effects of selenium (Se) on the growth, accumulation and possible mechanisms of Se transport in certain parts (roots, leaves, stamp and apex) of nettle (*Urtica dioica* L.) plants. Se was supplemented by one-shot and two repeated doses to the soil (2.0 and 4.0 mg Se per kg of substrate). Selenium content in roots increased linearly with dose and was significantly higher compared to other plant parts of interest. However, growth of the above-ground parts of plant as well as roots was slightly inhibited with increasing selenium concentration in comparison to the untreated plants. The content of phytochelatin2, a low molecular mass peptide containing a sulfhydryl group, correlated well with the Se content. This suggests a possible stimulation of synthesis of this plant peptide by Se.

## 1. Introduction

Selenium (Se) is present in the environment in elemental form or in the form of selenide (Se^2−^), selenate (SeO_4_^2−^), or selenite (SeO_3_^2−^). It is widely distributed in the Earth’s crust at concentrations averaging 0.09 mg kg^−1^ and in trace quantities in most plant and animal tissues [1]. Selenium is not classified as an essential plant nutrient, but it is important for human and animal nutrition [1,2]. Plants are capable of Se accumulation and transformation of the toxic inorganic forms into different chemical species, and thus can be used for Se-phytoremediation [3,4]. Selenate uptake through the root plasma membrane is mediated by the high-affinity sulfate transporter encoded by the *SHST1*, *SHST2*, and *SHST3* genes [5]. The expression of these root membrane transporters is negatively regulated by reduced glutathione (GSH) and/or sulfate (SO_4_^2−^). On the other hand, it is positively regulated by *O*-acetylserine because the latter is a precursor of cysteine and a product of nitrate assimilation [6]. The problems of transport, biochemistry, phytovolatilization, and phytoremediation of Se in higher plants have been reviewed by Terry and coworkers [4]. More recent papers show the suitability of transgenic plants for hyperaccumulation of Se from the environment [7–9]. Meta-analysis can be also used to suggest strategies for remediation of soils contaminated by various elements, including selenium [10].

Plants of the species *Astragalus*, *Stanleya*, *Morinda*, *Neptunia*, *Oonopsys*, and *Xylorhiza* are assumed to be Se-accumulators able to accumulate from hundreds to several thousands of mg of selenium per kilogram of dry weight. These plants grow readily on seleniferous soils. Crop plants and species of the genera *Distichlis* or *Atriplex* are Se-non-accumulators and grow on nonseleniferous soils (usually these can accumulate only 0.1 to 1 mg Se/kg dry weight, however maximum accumulated amounts of Se of about 25 mg Se/kg dry weight have been reported) [4]. Additionally, plant species which are referred to as secondary Se-accumulators (*Astragalus*, *Aster*, *Castilleja*, *Comandra*, *Grayia*, *Brassica* and others) are plants growing on soils with low selenium content yet able to accumulate high concentrations of the element (1,000 mg Se/kg dry weight) [11]. Moreover, has been shown that selenite contributes to a larger accumulation of non-protein thiol compounds in the roots and selenate contributes to their accumulation in the shoots [12]. Reduced glutathione (GSH) belongs to the group of important non-protein -SH-rich molecules. This important antioxidant plays a role in the detoxification of a variety of electrophilic compounds and peroxides. The process is catalyzed by glutathione *S*-transferases and glutathione peroxidase [13,14]. GSH is highly reactive and often found conjugated via its sulfhydryl moiety to other molecules such as NO (*S*-nitrosoglutathione). In addition, it can be used for synthesis of phytochelatins [compounds with a basic formula (γ-Glu-Cys)_n_-Gly (n = 2 to 11)] participating in the detoxification of heavy metals in plants. Phytochelatins have the ability to bind heavy metal ions via the SH groups of cysteine units and transport them to vacuole, where any immediate toxicity does not endanger the organism [15–19]. Plant cells display other ways to detoxify heavy metals in addition to phytochelatins [20].

The nettle (*Urtica dioica* L.) is a perennial plant growing in temperate and tropical wasteland areas around the world. The plant was naturalized in Brazil and other parts of South America. The maximum typical height of this plant species ranges from 2 to 4 meters. It produces pointed leaves and white to yellowish flowers. In folk medicine nettles have been used as a diuretic agent and to treat arthritis and rheumatism. Nowadays nettle is an important medical herb and consumed as a component of the human diet due to its content of minerals, chlorophyll, amino acids, lecithin, carotenoids, flavonoids, sterols, tannins and vitamins. Furthermore the roots of this plant contain other biological active compounds such as scopoletin, sterols, fatty acids, polysaccharides and isolectins. Several compounds present in the nettle have demonstrated antiviral properties potentially applicable for treatment of certain diseases e.g., HIV [21], and several common respiratory viruses [22]. Some compounds have also anti-proliferative effect on human prostate cancer cells [23].

The aim of this study was to investigate the effect of selenium on the growth and the Se accumulation in the certain parts (roots, leaves, stamp and apex) of nettle plants and the possible mechanism(s) of Se transport Furthermore, a possible mechanism of Se transport was suggested.

## 2. Experimental Section

### 2.1. Chemicals

Sodium selenite and other analytical grade reagents were purchased from Sigma Aldrich Chemical Corp. (St. Louis, MO, USA). Water used for preparation of solutions was demineralised by reverse osmosis using Aqua Osmotic 02 (Aqua Osmotic, Tišnov, Czech Republic) and further purified by Millipore RG (18 MΩ Millipore Corp., Billerica, MA, USA). Standard 10 mg/mL stock solutions of selenium were prepared by dissolving sodium selenate in water and stored in dark at 4 °C. The working standard solutions were prepared daily by dilution of the stock solutions.

### 2.2. Plant Material and Cultivation

Grown-up plants of the dioecious nettle (*Urtica dioica L.*) were used as a primary plant material. Nettle plants were removed from the soil and then green above-ground parts of the individual plants were cut and remaining rhizomes were rinsed with water. Twelve cuttings (2–3 centimeters long) were prepared from the rhizome and the surface of each cut was treated with a growth stimulator (AS–1, 0.06% nicotinic acid; 0.06% potassium α-naphtholacetate, Czech Republic). Modified cuts were planted in a mixture of garden soil and coarser river sand (1:1) for 20 days. After the root system developed (as soon as the first leaves appeared above the soil surface) the plants were singly placed in prepared plant boxes. River sand (3–4 cm) and 2,100 g of a mixture of garden soil and coarser river sand (1:1) were deposited in layers into each plastic plant box (15 cm × 15 cm × 15 cm). The plant boxes with young plants were placed in free space in an aviary covered with polyethylene sheet. Garden substrates used in this study were purchased from AGRO a.s. (Česká Skalice, Czech Republic). The substrate contained following essential nutrients: 275 mg N, 165 mg P_2_O_5_ and 425 mg K_2_O per litre. Declared pH value of the garden soil aqueous extract was 6. After three days acclimatization of the replanted plants, 5 mL (2 mg Se) or 10 mL (4 mg Se) Na_2_SeO_3_ were applied to each plant box according the scheme shown in Table 1. Adequate soil wetness was maintained by periodic watering. After 77 days (from selenium application) plants were carefully removed from the plant boxes and their above-ground and root parts were separated. The above-ground part was divided into an apex, younger leaves (first ten leaves from the apex), older leaves and a stalk. Underground parts were carefully cleaned of soil and washed on a sieve with flowing tap water and then with distilled water and placed on a filter paper to dry.

### 2.3. Sample Preparation for Fresh and Dry Weight Analysis

The plants were weighed on Sartorius R160P balances (Sartorius GmbH, Goettingen, Germany) immediately after collecting from the filter paper. Plant material was dried to the constant weight with a Premed evaporator (KBC G-100/250, Warszawa, Poland) and weighed. Dried samples were milled to a fine-grained powder (Ika A11 basic, Germany).

### 2.4. Sample Preparation for Selenium Determination

Mineralization of samples (0.01–0.30 g of the plant powder) was done using a MSL 1,200 microwave kiln (Milestone, Italy). Five millilitres of 65% nitric acid (max. 0.02 ppm Se) and one millilitre of 31% hydroperoxide were added to the plant powder. The plant powder was digested by an ETHOS SEL microwave digestion furnace (Milestone S.r.l, Italy) using a MDR 300/10 module. Decomposition was run according to the following program: 00:00–01:00 min, 250 W; 01:00–03:00 min, 0 W; 03:00–08:00 min, 250 W; 08:00–13:00 min, 400 W; 13:00–16:00 min, 500 W. Mineralized plant material was quantitatively transferred into the volumetric flasks and diluted by water up to10 mL.

### 2.5. Determination of Selenium Using Graphite Furnace Atomic Absorption Spectrometry

A UNICAM series M atomic absorption spectrometer with a graphite cuvette heated by an electric resistance coupled with a FS 95 autosampler (UNICAM) was used for selenium analysis. Ni(NO_3_)_2_ was used as a modifier. Zeeman correction in combination with correction via deuterium discharge lamp was used for background correction D2. The wavelength used (196.0 nm) was separated out from selenium lamp light to get the highest sensitivity.

### 2.6. Preparation of Plant Tissues for Thiol Determinations

Weighed plant tissues (approximately 0.2 g) were transferred to a test-tube and prepared according to Supalkova *et al.* [24]. Then, liquid nitrogen was added to the test-tube, and the samples were frozen to disrupt the cells. Subsequently, 1,000 μL of 0.2 M phosphate buffer (pH 7.2) was added to the test-tube. The mixture was prepared using an ULTRA-TURRAX T8 hand-operated homogenizer (IKA, Germany) at 25,000 rpm for 3 minutes. The homogenate was transferred to a new test-tube. The homogenate was shaken on a Vortex-2 Genie (Scientific Industries, New York, NY, USA) at 4 °C for 30 min. The homogenate was centrifuged (14,000 *g*) for 30 min at 4 °C using a Universal 32 R centrifuge (Hettich-Zentrifugen GmbH, Tuttlingen, Germany). The supernatant was filtered through a membrane filter (0.45 μm nylon filter disk, Millipore, Billerica, MA, USA) prior to analysis.

### 2.7. Determination of Thiols

The high performance liquid chromatography with electrochemical detection (HPLC-ED) system consisted of two solvent delivery pumps operating in the range of 0.001–9.999 mL/min (Model 582 ESA Inc., Chelmsford, MA, USA), a Metachem Polaris C18A reverse-phase column (150.0 × 2.1 mm, 5 μm particle size; Varian Inc., Santa Clara, CA, USA) and a CoulArray electrochemical detector (Model 5600A, ESA). The electrochemical detector includes three flow cells (Model 6210, ESA). Each cell consists of four analytical cells containing a carbon porous working electrode, two auxiliary and two reference electrodes. Both the detector and the reaction coil/column were thermostated. The optimal conditions were as follows: a gradient profile for simultaneous thiol separation starting at 100:0 (80 mM TFA-methanol), kept constant for 9 min, then decreasing to 85:15 during one minute, kept constant for 8 min, and finally increasing linearly up to 97:3 from 18 to 19 min, mobile phase flow rate of 0.8 mL/min, and column temperature of 40 °C. The sample (5 μL) was injected using an autosampler (Model 540 Microtiter HPLC, ESA). Further details are described in Diopan *et al.* [25].

### 2.8. Descriptive Statistics

Data were processed using MICROSOFT EXCEL® (USA) and STATISTICA.CZ Version 8.0 (Czech Republic). Results are expressed as mean ± standard deviation (S.D.) unless noted otherwise (EXCEL®). Statistical significances of the differences between Se content in treated and control plants were determined using STATISTICA.CZ. Differences with p < 0.05 were considered significant and were determined by using of one way ANOVA test (particularly Scheffe test), which was applied for means comparison.

## 3. Results and Discussion

### 3.1. Selenium Influence on Plants

Selenium content in plants is closely associated with the content of this element in soil and, thus, influences the amounts of selenium in foodstuffs of both plant and animal origin. It is necessary to assure sufficient amounts of selenium, which is indispensable for the good health of farm animals, in fodder. Transport of Se in a plant depends on many factors, e.g., plant genotype, presence of sulfur and others [11]. Selenium in small amounts stimulates plant growth but at higher levels it causes toxicity manifest as suppression of growth and the appearance of chlorosis on leaves [26].

### 3.2. Plant Behavior

**Figure 1.**
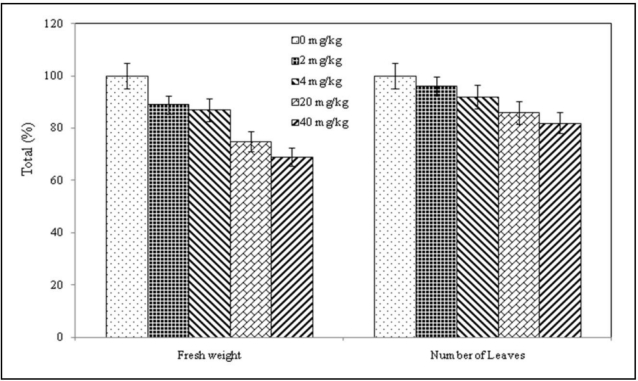
Number of leaves and fresh weight of the plants treated with 0, 2, 4, 20 and 40 mg Se per kg of substrate.

The influence of selenium on a plant behavior and Se accumulation in the nettle were investigated. We applied two different doses of selenium to the soil (2.0 and 4.0 mg Se per kg of substrate) according to the Table 1. At the beginning of the experiment, a decrease of plant growth with different applied Se doses was noticed (samples 2, 3, 4 and 5). This growth decrease was indicated by considerable growth moderation up to the point of plant growth stagnancy in comparison with control plants. Marked plant growth moderation was observed between 5 and 10 days after a one-shot selenium dose (samples 2, 3, 4 and 5; Table 1). The decreased growth stopped on the twentieth day of the experiment. This phenomenon was observed in all Se treated samples. On some leaves, chlorosis of laminas of lower plant areas exposed to higher Se dose (samples 3 and 5; 4 mg Se; Table 1) with formation of irregular flavescent occurred. The leaves subsequently fell off. The expression of chlorosis, a pathological state induced by toxic Se dose resorption, was masked in part by inherent pigment changes related to the plants’ life cycle that also appeared in the control plants (they turn yellow and fall off one or two leaf levels in connection with the end of vegetative period). The number of leaves and fresh weight of the experimental plants are shown in Figure 1.

### 3.3. Root System of Plants

It was found out that root system volume and segmentation of the experimental plants was dependent on the amount of selenium applied to the soil. Natural root development was suppressed by increased Se concentration in the soil. The most massive and the most divaricated root system was observed in the control plants (100%). Decreases of the fresh weight of roots to 86% (2 mg/kg) and 52% (4 mg/kg) appeared in plants exposed to one-shot Se doses (samples 2 and 3). In the case of repeated dosages the root weights decreased to 59% (10 dose of 2 mg Se/kg) and 46% (10 doses of 4 mg Se/kg) compared to the control (not shown). Segmentation, divaricating and overall profuseness (small roots representation) decreased proportionally with the amount of Se applied which could be in contradiction with the overall plant behavior (sample 5, show unwent the biggest total Se addition but showed the fastest consolidation after grow depression and further the fastest growth and above-ground part progression in comparison with other Se supplemented samples).

### 3.4. Selenium Content in Plant

Atomic absorption spectrometry using the hydride technique is a commonly used method for selenium quantification [27,28]. Selenium determination in a graphite cuvette using Ni(NO_3_)_2_ as the modifier was used in this study. Samples were placed five times in the cuvette, where the evaporation and atomization occurred. The detection limit of the method was 180 pg of selenium on 1 g of dry weight (DW), with a relative standard deviation of 5%. A calibration curve was strictly linear in a range from 5 to 50 ng/mL (y = 0.0019x + 0.0026, R^2^ = 0.9966).

**Figure 2.**
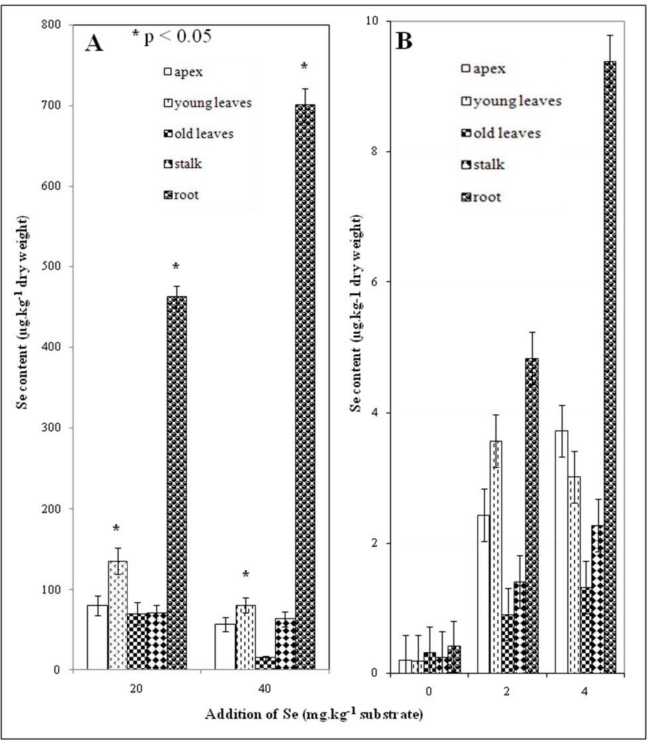
Selenium content in individual parts (apex, young leaves, old leaves, stalk and root) of nettle plants treated with 20 and 40 mg Se/kg (A); 2 and 4 mg Se/kg (B). Other experimental conditions as described in the Experimental section.

Selenium content in roots increased linearly with the amount of Se applied (Figure 2). Growth of above-ground plant parts and root stagnated in connection with increasing selenium concentration. Selenium concentrations in roots of all treated plants significantly exceed the amounts in other plant tissues (Figure 2). Application of 20 mg Se per kg of soil resulted in considerable a selenium content increase in all above-ground plant parts (Figure 2A). Lower Se content in the above-ground parts of plants was observed for the addition of 40 mg Se per kg of soil in comparison with application of 20 mg Se/kg (Figure 2A). This effect is probably connected with the higher expression of metal-binding proteins such as glutathione and phytochelatins [25,29,30]. In c ase of one-shot doses, equal selenium concentrations were found in plants treated with 2 mg Se/kg (variant 2) as in plants treated with 4 mg Se/kg (Figure 2B).

The determined selenium concentration in above-ground plant parts proves that inorganic selenium is intensively transported from underground plant parts by the xylem to photosynthetically active organs and further that selenium is probably incorporated to organic species. Photosynthesis products (reserve materials and indispensable trace elements) are accumulated in reserve rhizome tissues. Higher concentrations were determined in the roots of treated plants. Significant differences in selenium content were determined between older and younger leave tissues (Figure 2). Inorganic selenium is probably transported to younger leaves, where it is incorporated into metal-binding proteins and therefore the concentration in older leaves decreases.

### 3.5. Low Molecular Mass Thiols–phytochelatins

**Figure 3.**
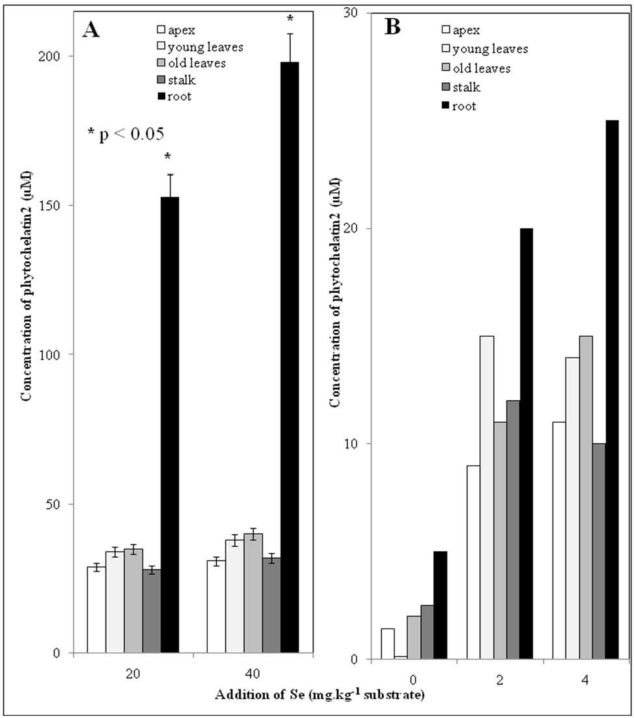
Phytochelatin2 (PC2) content in individual parts (apex, young leaves, old leaves, stalk and root) of nettle plants treated with 20 and 40 mg Se/kg (A); 2 and 4 mg Se/kg (B). Other experimental conditions as described in the Experimental section.

There is increasing evidence of the close connection between Se uptake and enhanced synthesis of phytochelatins (PCs) [12,31–33]. PCs as heavy metal detoxifying peptides of plant origin are of a great interest. Plant tissues were prepared according to the protocol given in the Experimental section. The prepared plant material was further analyzed by HPLC-ED. Electrochemical detection was shown to be suitable for detection of plant low molecular mass thiols [18,25,29,30,34]. Using HPLC-ED, the content of phytochelatin2 (PC2) was determined in all plant parts of interest (apex, young and old leaves, stalk and root). The results are shown in Figures 3A and 3B. The content of PC2 increased with increasing Se dose. The highest content was determined in roots of plants treated with the highest dose of Se, which corresponds well with the Se content in this plant part shown in Figure 2A. The content of PC2 in the roots of plants treated with the second highest dose of Se was also significantly higher compared to control. It clearly follows from the results obtained that applied Se stimulates the synthesis of low molecular mass thiols such as the as phytochelatins, which could be promising for possible phytomining of this element and/or phytoremediation of soils contaminated by Se.

## 4. Conclusions

Several nettle extracts are used for the preparation of products used to cure many diseases. Based on the obtained results selenium is accumulated in different parts of treated plants, but mostly in roots, followed by young leaves and other plant organs. For this reason selenium treated plants, particularly their new leaves and roots, could be useful for the preparation of new pharmaceutical drugs with higher selenium content.

## Figures and Tables

**Table 1.** Experimental scheme.

**Sample ID**		**Applied dose [mg Se/kg soil]**	**Number of doses**	**Total Se addition [mg Se/kg soil]**
A	1	0.0	0	0.0
	2	2.0	1	2.0
	3	4.0	1	4.0
B	4	2.0	10	20.0
	5	4.0	10	40.0
